# Identification of the YfgF MASE1 domain as a modulator of bacterial responses to aspartate

**DOI:** 10.1098/rsob.130046

**Published:** 2013-06

**Authors:** Melissa Lacey, Agnieshka Agasing, Rebecca Lowry, Jeffrey Green

**Affiliations:** Department of Molecular Biology and Biotechnology, The Krebs Institute, University of Sheffield, Sheffield S10 2TN, UK

**Keywords:** chemotaxis, cyclic-di-GMP, MASE1 domain, oxidative stress, *Salmonella*

## Abstract

Complex 3′-5′-cyclic diguanylic acid (c-di-GMP) responsive regulatory networks that are modulated by the action of multiple diguanylate cyclases (DGC; GGDEF domain proteins) and phosphodiesterases (PDE; EAL domain proteins) have evolved in many bacteria. YfgF proteins possess a membrane-anchoring domain (MASE1), a catalytically inactive GGDEF domain and a catalytically active EAL domain. Here, sustained expression of the *Salmonella enterica* spp. Enterica ser. Enteritidis YfgF protein is shown to mediate inhibition of the formation of the aspartate chemotactic ring on motility agar under aerobic conditions. This phenomenon was c-di-GMP-independent because it occurred in a *Salmonella* strain that lacked the ability to synthesize c-di-GMP and also when PDE activity was abolished by site-directed mutagenesis of the EAL domain. YfgF-mediated inhibition of aspartate chemotactic ring formation was impaired in the altered redox environment generated by exogenous *p-*benzoquinone. This ability of YfgF to inhibit the response to aspartate required a motif, ^213^Lys-Lys-Glu^215^, in the predicted cytoplasmic loop between trans-membrane regions 5 and 6 of the MASE1 domain. Thus, for the first time the function of a MASE1 domain as a redox-responsive regulator of bacterial responses to aspartate has been shown.

## 2. Introduction

3′-5′-cyclic diguanylic acid (c-di-GMP) is a bacterial second messenger that regulates diverse cellular functions including the cell cycle, synthesis of extracellular polysaccharide and virulence factors, and transitions between sessility and motility [1]. Generally, motility and the production of virulence factors are promoted by low intracellular concentrations of c-di-GMP, whereas high intracellular concentrations promote biofilm formation and a sessile lifestyle [2]. In many bacteria, c-di-GMP concentrations are controlled by complex networks of diguanylate cyclases (DGC; GGDEF domain proteins) and phosphodiesterases (PDE; EAL or HD-GYP domain proteins). Coordination of the opposing DGC and PDE activities that form these networks is required for optimal bacterial responses to environmental change [1]. This is achieved, at least in part, via signal perception and/or signal transduction domains, such as BluF (blue-light sensory domain), HAMP (histidine kinases–adenylyl cyclases–methyl accepting chemotaxis proteins–phosphatase domain), GAF (cGMP PDE–adenylyl cyclase–FhlA domain), PAS (Per-Amt-Sim domain), as well as DNA-binding domains and as yet uncharacterized domains, that are known to, or thought to, control the DGC and PDE activities of the corresponding proteins [1].

Facultative anaerobic enterobacteria such as *Salmonella enterica* spp. Enterica ser. Enteritidis (*S*. Enteritidis) experience transitions between aerobic and anaerobic environments during their pathogenic and commensal lifestyles. In these bacteria, the *yfgF* gene encodes a c-di-GMP PDE that is maximally expressed under anaerobic conditions in *Escherichia coli,* with expression under microaerobic conditions (2.5% oxygen atmosphere) being only slightly reduced compared with that observed under anaerobic conditions [3]. The *yfgF* promoter of *S*. Enteritidis is very similar to that for *E. coli*, retaining both the FNR box at −41.5 (TTGAT––ATCAA in *E. coli*; TTGAC––ATCAA in *S*. Enteritidis) and the −10 element (TAGAAT in *E. coli*; TACAAT in *S*. Enteritidis), suggesting the presence of a Class II FNR-dependent promoter in both bacteria [3]. *Salmonella* Enteritidis YfgF has three domains: a trans-membrane MASE1 (membrane-associated sensor 1) domain; a degenerate (inactive) GGDEF domain; and an active EAL domain [3]. While GGDEF and EAL domains are associated with well-defined functions, MASE1 domains are poorly characterized. The MASE1 domain was identified as a trans-membrane domain of unknown function often associated with histidine kinase, GGDEF, GGDEF-EAL and PAS domains [4], implying that MASE1 domains act as sensors that modulate the activity of various output domains.

The starting point for this work was the hypothesis that the MASE1 membrane anchor acts as a receptor for a signal that modulates YfgF function. It is shown that: (i) the PDE activity of *S*. Enteritidis YfgF (STM2503 in *S. enterica* ser. Typhimurium) restores motility to a c-di-GMP PDE-deficient strain of *E. coli* (*yhjH* mutant); (ii) sustained expression of *yfgF* under aerobic conditions specifically inhibits formation of the *S*. Enteritidis aspartate chemotactic ring independently of c-di-GMP and PDE activity; (iii) YfgF-mediated inhibition of the bacterial response to aspartate is relieved under anaerobic conditions and by the presence of exogenous *p*-benzoquinone under aerobic conditions; and (iv) amino acid residues Lys-213, Lys-214 and Glu-215 of a predicted cytoplasmic loop located between trans-membrane regions 5 and 6 of the MASE1 domain are required for YfgF function. Thus, it was concluded that YfgF affects bacterial behaviour in c-di-GMP-dependent (through the action of its PDE activity to modulate motility) and -independent (through MASE1 domain-mediated changes in the response to aspartate) mechanisms.

## 3. Material and methods

### 3.1. Bacterial strains, plasmids and growth conditions

The prototroph *S*. Enteritidis 3934 and a derivative *Salmonella ΔXII* which has deletions of all 12 genes encoding GGDEF proteins [5] were used in the investigation of c-di-GMP-independent regulation of aspartate chemotaxis by YfgF. Sustained expression of the *S*. Enteritidis YfgF protein (and site-directed variants thereof) was achieved by expression of the corresponding genes under the control of the pBAD promoter in the indicated plasmids (all YfgF proteins were expressed with a C-terminal FLAG-tag for immunoassays). The pGS2421 plasmid that encoded the wild-type *yfgF* gene was the template for site-directed mutagenesis using the Quikchange (Stratagene) protocol with appropriate oligonucleotides to express the YfgF variants studied here. The prototroph *Escherichia coli* K-12 MG1655 and an isogenic *yhjH* mutant (JRG5963/HSG139) [6] were used to demonstrate that the plasmid-encoded *yfgF* genes were expressed and resulted in the synthesis of folded functional proteins. Bacteria were routinely cultured in L broth [7] supplemented with ampicillin (100 μg ml^−1^) when necessary. DNA was isolated and manipulated by conventional methods [8].

### 3.2. Motility assays for chemotaxis

Motility was determined using motility agar (0.5% tryptone, 0.5% NaCl, 0.3% agar) as described previously [3], or minimal semi-solid agar [9] using Evans defined medium [10] containing 100 mM chemoattractant, as indicated. Motility in aspartate gradients generated independently of nutrient consumption was measured by spotting bacterial suspensions grown under aerobic or anaerobic conditions onto Evans minimal medium without a carbon source approximately 20 mm from a 6 mm diameter filter disc loaded with 10 µl of 1 M aspartate. The plates were then incubated under aerobic or anaerobic conditions, as indicated. Ampicillin (100 μg ml^−1^), arabinose (0.0002% or 0.002% w/v for *E. coli* and *S*. Enteritidis plates, respectively) and *p*-benzoquinone (200 μg ml^−1^) were added as required. Motility plates were inoculated with a stab from agar stock plate and incubated at 37°C (*E. coli*) or 28°C (*S*. Enteritidis). Identification of the aspartate ring by perturbation was achieved by adding 2 µl of 1 M aspartate 5 mm ahead of the motility halo on a motility plate, which was then incubated for 8 h [11].

### 3.3. Immunoblotting

Samples were collected from cultures (50 ml) grown at 28°C in motility broth (0.5% tryptone, 0.5% NaCl) to an optical density at 600 mm of 0.1–0.2. Where indicated, *p*-benzoquinone (200 μg ml^−1^) was then added and incubated for 1 h at 28°C. The cell pellets were boiled in reducing Laemmli sample buffer with 8 M urea, before electrophoresis on sodium dodecylsulphate-polyacrylamide gels [12] followed by electroblotting onto Hybond-C membrane (Amersham). Proteins were detected using anti-FLAG primary antibody raised in rabbit (1 : 30 000) followed by anti-rabbit immunoglobulin-horseradish peroxidase conjugate (1 : 10 000 dilution; Amersham). Bound antibody was visualized by chemiluminescence (ECL detection kit, Amersham).

## 4. Results

### 4.1. YfgF inhibits the development of the aspartate chemotactic ring in *S*. Enteritidis independently of c-di-GMP under aerobic conditions

**Figure 1.**
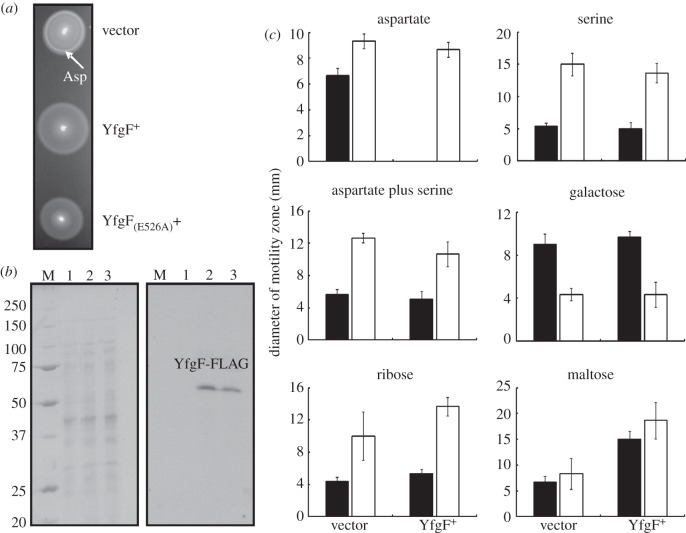
*S*. Enteritidis YfgF modulates aerobic aspartate chemotaxis via a c-di-GMP-independent mechanism. (*a*) Swimming motility of *S*. Enteritidis transformed with plasmids expressing no YfgF (vector control), *S*. Enteritidis YfgF and *S*. Enteritidis YfgF_(E526A)_. The aspartate (Asp) chemotactic ring is arrowed. (*b*) Coomassie blue-stained SDS polyacrylamide gel (left) and Western blot developed with anti-FLAG antibodies (right) for *S*. Enteritidis transformed with pGS2390 (vector control, lanes 1), pGS2421 (YfgF^+^, lane 2), or pGS2460 (YfgF_(E526A)_, lane 3). The Coomassie blue-stained gel shows the protein loading for each lane. The locations of the YfgF protein and the protein markers to calibrate the gel (lane M; molecular weights in kDa) are indicated. (*c*) Effects of sustained expression of YfgF. The chemotactic response of *S*. Enteritidis was assessed on media containing the indicated chemoeffector(s) under aerobic (black bars) and anaerobic (white bars) conditions. Each experiment was performed a minimum of three times; the mean values and standard deviations are shown.

When *S*. Enteritidis transformed with the vector control (pGS2390) was incubated on motility agar plates, two chemotactic rings were observed (figure 1*a*). The innermost ring was assigned as the aspartate ring (arrowed in figure 1*a*) and the outermost ring as the serine ring [11,13]. However, the aspartate chemotactic ring was not observed when *S*. Enteritidis was transformed by plasmid pGS2421 to permit sustained expression of YfgF (with a C-terminal FLAG tag; figure 1*a*). Expression of YfgF from pGS2421 in the *S.* Enteritidis derivative *Salmonella ΔXII*, which lacks all GGDEF proteins and contains no detectable c-di-GMP [5], also inhibited the development of the aspartate chemotactic ring (not shown).

The *Salmonella ΔXII* strain lacks GGDEF domain proteins and detectable c-di-GMP, and thus the PDE activity of YfgF is unlikely to account for the inhibition of aspartate chemotaxis. Accordingly, a YfgF variant, YfgF_(E526A)_, that lacks PDE activity was still able to prevent the appearance of the aspartate chemotactic ring in both wild-type *S*. Enteritidis (figure 1*a*) and *Salmonella ΔXII* (not shown). The abolition of PDE activity by the E526A substitution was demonstrated by the inability of YfgF_(E526A)_ to degrade c-di-GMP *in vitro* and its failure to complement the motility defect of an *E. coli yhjH* mutant *in vivo* (not shown). Western blotting using anti-FLAG-tag antibodies indicated that the YfgF-FLAG and YfgF_(E526A)_-FLAG proteins were expressed at similar levels in the transformed bacteria (figure 1*b*); note that, like many other membrane proteins, YfgF-FLAG (molecular mass 88.5 kDa) migrates aberrantly on denaturing polyacrylamide gels (*M*_r_ ∼ 65 kDa).

The extent of the influence of YfgF on chemotaxis was measured using minimal media containing either a single chemoattractant or a combination of two chemoattractants such that consumption of the substrate(s) generates concentration gradients. Expression of YfgF-FLAG in *S*. Enteritidis under aerobic conditions abolished colony expansion in the presence of aspartate, but did not affect the responses to serine, galactose, ribose or maltose (figure 1*c*, black bars). Furthermore, YfgF did not impair the chemotactic response to serine even in the presence of aspartate (figure 1*c*). Under anaerobic conditions both strains responded to all the chemoeffectors tested (figure 1*c*, white bars), including a positive response to aspartate. Expression of YfgF-FLAG in *S.* Enteritidis under aerobic conditions had no effect on the response to the repellants nickel and cobalt (data not shown). Together these data suggest that YfgF modulates colony expansion of *S*. Enteritidis in response to aspartate under aerobic conditions independently of c-di-GMP.

### 4.2. Sustained expression of YfgF inhibits bacterial migration toward a source of aspartate under aerobic conditions

**Figure 2.**
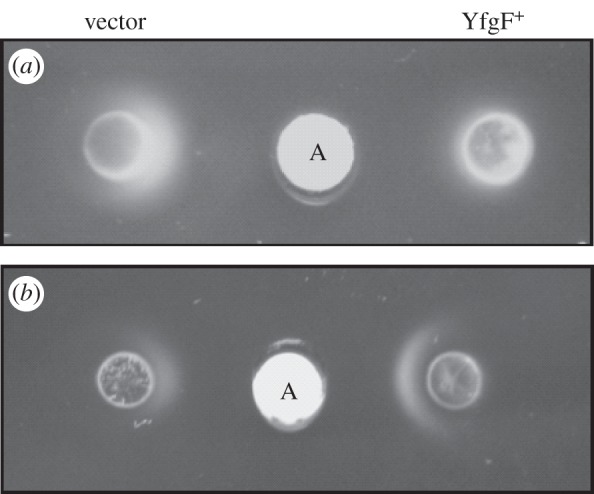
Sustained expression of YfgF inhibits the migration of *S*. Enteritidis towards a source of aspartate. An aspartate concentration gradient was created by placing a filter disc (marked as A in the figure) loaded with 10 µl of 1 M aspartate in the centre of a minimal medium agar plate lacking other carbon sources. Suspensions of *S*. Enteritis transformed with pGS2390 (vector control) or pGS2421 (YfgF^+^) were pipetted onto the agar, and the plates were incubated under (*a*) aerobic and (*b*) anaerobic conditions at 28°C for 16 h. Chemotaxis was visible as the crescent formed as the bacteria migrated from the point of inoculation towards the source of aspartate.

In the experiments described in §4.1, the formation of the aspartate chemotactic ring is dependent upon the ability of the bacteria to consume aspartate and thereby create a concentration gradient. In such experiments YfgF might impair bacterial migration by inhibiting aspartate utilization rather than by inhibiting aspartate chemotaxis. Therefore, the effects of sustained YfgF-FLAG expression on the ability of *S*. Enteritidis to migrate in an aspartate gradient was tested on motility agar in which a concentration gradient was formed by diffusion from a filter disc loaded with aspartate placed on an agar plate. The experiment showed that *S*. Enteritidis transformed with the vector (pGS2390) was able to migrate towards the aspartate-loaded disc, whereas *S*. Enteritidis expressing YfgF-FLAG failed to do so under aerobic conditions (figure 2*a*). In contrast, under anaerobic conditions the inhibition of aspartate chemotaxis by YfgF-FLAG was not observed (figure 2*b*), confirming that YfgF is an inhibitor of bacterial migration in response to aspartate only under aerobic conditions (figure 1*c*). This experiment indicates that the metabolic generation of aspartate gradients is not necessary for the aerobic inhibition of aspartate chemotactic ring formation by YfgF.

### 4.3. The ability of YfgF to inhibit the formation of the aspartate chemotactic ring is modulated by exogenous *p*-benzoquinone

**Figure 3.**
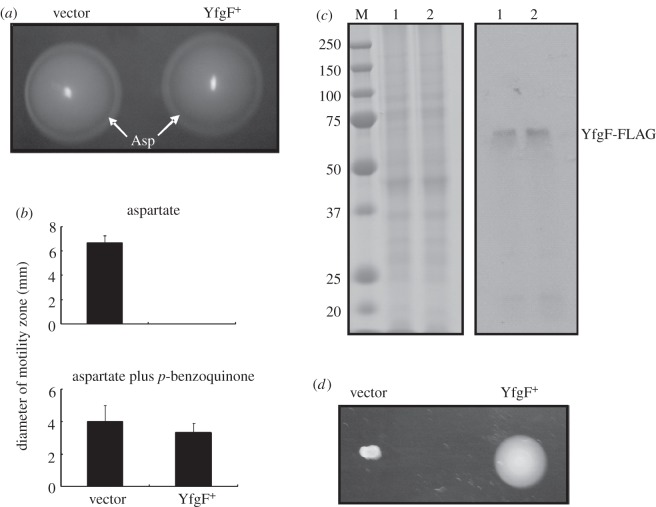
YfgF-mediated inhibition of aspartate chemotaxis is relieved by exogenous *p*-benzoquinone. (*a*) Swimming motility of *S*. Enteritidis transformed with plasmids expressing no YfgF (vector control) and *S*. Enteritidis YfgF in the presence of *p*-benzoquinone (200 µg ml^−1^) under aerobic conditions. The aspartate (Asp) chemotactic ring (arrowed) is apparent in the absence (left) and presence (right) of YfgF. (*b*) The motility of *S*. Enteritidis was assessed on medium containing aspartate in the presence and absence of *p*-benzoquinone under aerobic conditions. In all cases, each experiment was performed a minimum of three times (mean values with standard deviation are shown). (*c*) Coomassie blue-stained SDS polyacrylamide gel (left) and Western blot developed with anti-FLAG antibodies (right) for *S*. Enteritidis transformed with pGS2421 (YfgF^+^) in the absence (lane 1) and presence (lane 2) of *p*-benzoquinone (200 µg ml^−1^). The Coomassie blue-stained gel shows the protein loading for each lane. The locations of the YfgF protein and the protein markers to calibrate the gel (lane M; molecular weights in kDa) are indicated. (*d*) Motility of *E. coli yhjH* mutant transformed with pGS2390 (vector control) or pGS2421 (YfgF^+^) in the presence of *p*-benzoquinone (200 µg ml^−1^). Plates were incubated under aerobic conditions for 20 h at 37°C.

In *E. coli* K-12 *yfgF* expression is activated by the oxygen-responsive transcription factor FNR and maximal under anaerobic conditions [3]. Analysis of the DNA sequence of the *S*. Enteritidis *yfgF* promoter region indicated that the FNR regulatory element is conserved. Despite *E. coli yfgF* expression being maximal under anaerobic conditions, the *yfgF* mutant exhibits an oxidative stress phenotype [3]. Furthermore, the YfgF-mediated inhibition of aspartate chemotaxis observed here was apparent under aerobic but not anaerobic conditions (figures 1*c* and 2). Therefore, the effect of modulating the redox environment on YfgF function was investigated. Addition of increasing concentrations of *p*-benzoquinone (which inhibits respiration, generates reactive oxygen species and lowers the proton motive force [13–15]) to motility plates resulted in restoration of the aspartate chemotactic ring under aerobic conditions (figure 3*a*). The identity of the aspartate ring was confirmed by spotting aspartate onto motility plates and observing colony morphology (not shown). Furthermore, aerobic colony expansion on minimal agar containing aspartate was restored in the presence of *p*-benzoquinone (figure 3*b*). Western blotting using antibodies to the FLAG-tag showed that the level of YfgF protein in the presence and absence of *p*-benzoquinone were similar (figure 3*c*) and the YfgF-FLAG protein was functional in the presence of *p*-benzoquinone as judged by the ability to complement the motility defect of an *E. coli yhjH* mutant (figure 3*d*). Therefore, it was concluded that a redox signal, generated by the presence of exogenous *p*-benzoquinone in the experiments described here, is perceived by YfgF to restore the aspartate chemotactic ring.

### 4.4. A motif (Lys-Lys-Glu) linking trans-membrane regions 5 and 6 is required for function of the YfgF MASE1 domain

**Figure 4.**
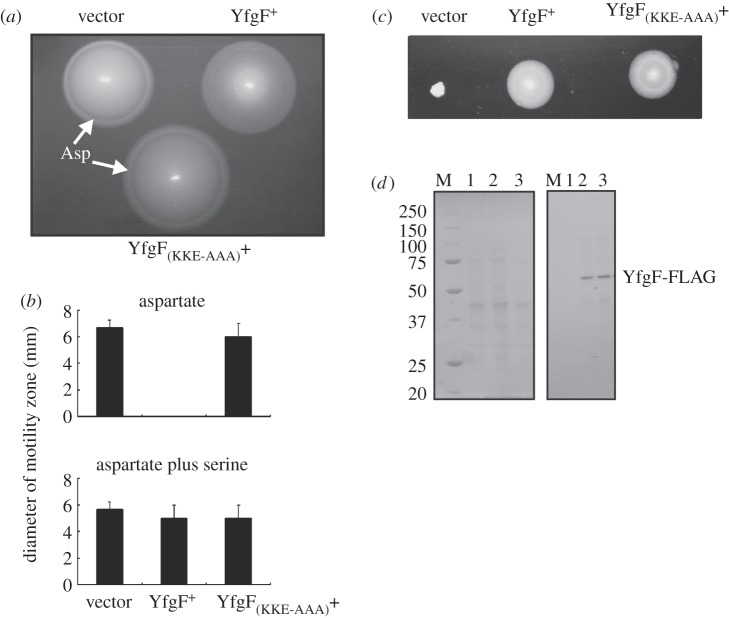
Replacement of Lys-Lys-Glu (amino acids 213–215) by Ala-Ala-Ala in the YfgF MASE1 domain permits formation of the aspartate chemotactic ring and *S*. Enteritidis colony expansion. (*a*) Swimming motility of *S*. Enteritidis transformed with plasmids expressing no YfgF (vector control, left), *S*. Enteritidis YfgF^+^ (right) and *S*. Enteritidis YfgF_(KKE-AAA)_^+^ (bottom). (*b*) Motility of *S*. Enteritidis on medium containing aspartate or aspartate plus serine was measured under aerobic conditions as described above. (*c*) YfgF_(KKE-AAA)_ complements the motility defect of an *E. coli* K-12 *yhjH* mutant. Swimming motility of *E. coli yhjH* transformed with pGS2390 (vector), pGS2421 (YfgF) and pGS2426 (YfgF_(KKE-AAA)_). Plates were incubated for 20 h at 37°C under aerobic conditions. (*d*) Coomassie blue-stained SDS polyacrylamide gel (left) and Western blot developed with anti-FLAG antibodies (right) for *S*. Enteritidis transformed with pGS2390 (vector control, lane 1), pGS2421 (YfgF^+^, lane 2), or pGS2426 (YfgF_(KKE-AAA)_, lane 3). The Coomassie blue-stained gel shows the protein loading for each lane. Lane M contains protein markers to calibrate the gel (molecular weights in kDa are indicated). The location of the YfgF protein is also indicated.

The *E. coli* YfeA protein has a similar domain structure to YfgF (MASE1-GGDEF-EAL), including a catalytically inactive GGDEF domain and catalytically active EAL domain. Expression of YfeA in *S*. Enteritidis did not affect the bacterial response to aspartate; in contrast, expression of *E. coli* YfgF (73% identical and 87% similar to *S.* Enteritidis YfgF over 733 amino acids) resulted in the same phenotypes as were observed for *S*. Enteritidis YfgF (not shown). These observations and the experiments described above suggested a possible function for the YfgF MASE1 domain as a redox sensor. Three cysteine residues (Cys-131, Cys-183 and Cys-231) within membrane spanning regions 4, 5 and 6 of YfgF were initially targeted for site-directed mutagenesis as they could mediate a redox signal by changes in oxidation state; however, the phenotypes of bacteria expressing these YfgF variants was the same as those expressing unaltered YfgF (i.e. aspartate chemotactic ring formation was inhibited; not shown). Alignment of the amino acid sequence of *S*. Enteritidis YfgF with the *E. coli* YfgF and YfeA MASE1 domains allowed the identification of residues that are conserved in *S*. Enteritidis and *E. coli* YfgF but are not in YfeA. Therefore, selected conserved charged/polar amino acids within membrane spanning regions 3, 7, 8 and 9 (Tyr-83, Glu-84, Thr-243, Asn-244, Thr-246, Leu-247, Ser-248, Arg-260, Ser-299, Ser-300, Ser-301, Tyr-302) were replaced, but these variants also exhibited wild-type behaviour (not shown). Thus, a third phase of site-directed mutagenesis was targeted towards conserved residues in the loops linking the trans-membrane regions. Replacement of Arg-109, Arg-110, His-115, Asp-117, Ser-151, Arg-191, Asn-192, Arg-197, Tyr-199, Tyr-200, Gln-202, Leu-203, Lys-204, Gln-206, Asp-208, Lys-264, His-279 showed that these residues were not required for YfgF-mediated inhibition of the response to aspartate; the phenotypes of all these variants were similar to wild-type YfgF (not shown). However, replacement of a motif Lys-Lys-Glu (positions 213–215, located in the cytoplasmic loop between trans-membrane regions 5 and 6; pGS2426) by Ala-Ala-Ala abolished the ability of YfgF to inhibit the formation of the aspartate chemotactic ring (figure 4*a*) and permitted colony expansion on aspartate-containing medium without affecting the response to serine (figure 4*b*). These data indicated that the integrity of the predicted cytoplasmic loop located between trans-membrane regions 5 and 6 was required for YfgF MASE1 function. Replacement of the Lys-Lys-Glu motif did not impair the ability of YfgF to complement the motility defect of an *E. coli yhjH* mutant, indicating that the overall fold and PDE activity of the mutant protein were retained (figure 4*c*). Furthermore, immunoblotting using anti-FLAG antibodies indicated that the expression of YfgF and YfgF_(KKE-AAA)_ were similar, and hence the inability of the latter to mediate a response to aspartate was not caused by inadequate expression (figure 4*d*).

## 5. Discussion

Here, it is shown that *S*. Enteritidis YfgF controls bacterial motility by c-di-GMP-dependent and c-di-GMP-independent mechanisms, as shown by PDE-dependent restoration of motility to an *E. coli yhjH* mutant, and PDE-independent, MASE1 domain-mediated inhibition of bacterial migration in response to aspartate. The results presented here used YfgF protein with a C-terminal FLAG-tag, but it should be noted that similar results were obtained with YfgF carrying a C-terminal His-tag (not shown), suggesting that the presence of the C-terminal tag does not impair YfgF function.

The initial experimental conditions required substrate (aspartate) utilization to create a concentration gradient and showed that YfgF specifically inhibited the formation of the aspartate chemotactic ring and expansion of bacterial colonies on aspartate medium without affecting the response to serine or sugar molecules under aerobic conditions (figure 1). When exposed to aspartate gradients generated independently of aspartate metabolism bacteria expressing *yfgF* were still unable to move along the concentration gradient (figure 2). Therefore, YfgF specifically inhibited *S*. Enteritidis colony expansion in response to aspartate in a c-di-GMP-independent manner under aerobic, but not anaerobic, conditions.

**Figure 5.**
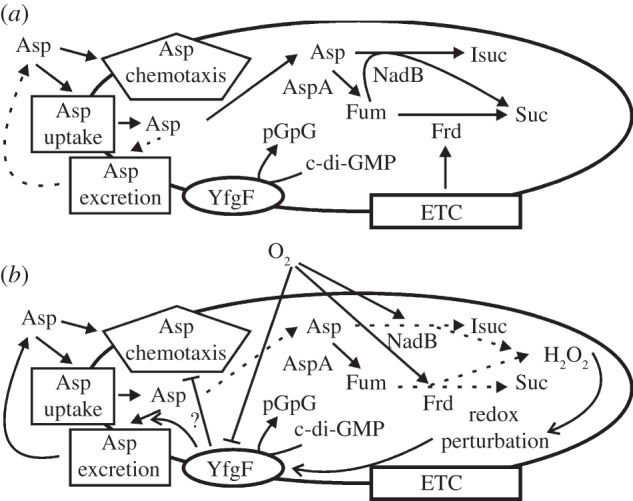
YfgF modulates aspartate chemotaxis to lessen oxidative damage when anaerobic bacteria are first exposed to molecular oxygen (O_2_). (*a*) In the absence of O_2_, expression of *yfgF* is maximal [3] and the proton motive force is low [24]. Under these conditions aspartate chemotaxis (in the presence of an appropriate energy source), aspartate uptake and metabolism are supported (solid arrows). Aspartase (AspA) converts Asp to fumarate (Fum), which in turn is reduced to succinate (Suc) by fumarate reductase (Frd) using reducing equivalents from the electron transport chain (ETC). Aspartate is also the substrate for NadB, which converts Asp to iminosuccinate (Isuc), which is required for NAD^+^ biosynthesis, with Fum acting as the electron acceptor [25]. (*b*) If left unchecked, when anaerobic bacteria are exposed to O_2_ NadB and Frd use O_2_ as an electron acceptor [22,23], generating the reactive oxygen species hydrogen peroxide (H_2_O_2_) (broken arrows), resulting in oxidative stress and redox perturbation. It is suggested (denoted by question mark) that the MASE1 domain of YfgF perceives the onset of this stress and either inhibits (⊥) aspartate chemotaxis or promotes (↑) aspartate excretion to decrease Asp utilization and thereby limit H_2_O_2_ generation until the bacteria have adapted to the new aerobic conditions. The presence of O_2_ switches off the transcription factor FNR, thereby minimizing *yfgF* expression (⊥). After adaptation growth and protein turnover removes YfgF from the now aerobic bacteria, restoring aspartate utilization and observable aspartate chemotaxis. Sustained expression of *yfgF* in the experimental system used here has allowed this normally transient behaviour to be revealed. Hydrolysis of c-di-GMP by YfgF promotes motility under anaerobic and aerobic conditions.

The ability of YfgF to inhibit aspartate chemotaxis was modulated by exogenous *p*-benzoquinone (figure 3), suggesting that YfgF activity responds to a redox signal. Site-directed mutagenesis identified a motif Lys-Lys-Glu (position 213–215) in the MASE1 domain that was required for YfgF-mediated inhibition of aspartate taxis (figure 4). This motif is likely to represent a component of a redox sensing and/or effector region of YfgF. The *E. coli* serine chemoreceptor Tsr is able to sense changes in the proton motive force, and it has been suggested that this might involve charged amino acids located on either side of the membrane spanning regions of the protein [16,17]. It is possible that the MASE1 amino acid motif identified here could form part of a similar sensory mechanism. However, it is not known whether the apparent inhibition of aspartate taxis by the YfgF MASE1 domain is direct or indirect. For direct regulation, it is not obvious how the cytoplasmic loop linking trans-membrane regions 5 and 6 of YfgF could effectively inhibit the periplasmic interactions between Tar and its effector, aspartate. However, apparent inhibition of aspartate taxis would be observed if YfgF acted to promote aspartate excretion from the bacterium. The excreted aspartate could then act as an attractant, and thereby oppose the outward migration of the colony. Such apparent inhibition of aspartate chemotaxis has been observed previously. Thus, some of the complex symmetrical patterns formed by motile *E. coli* cells on soft-agar plates were found to be associated with the response of Tar to aspartate excreted by the bacteria triggered in part by oxidative stress [18–20]. Subsequently,  *E. coli aer* (aerotaxis signal transducer) mutants supplied with succinate failed to migrate from the point of inoculation on semi-solid agar, but this was not the case for the *E. coli aer tar* double mutant [21]. These observations were rationalized by suggesting that under these conditions the bacteria excrete aspartate, which acts as an attractant constraining colony expansion in the absence of aerotaxis. Hence, it is possible that YfgF is the redox sensor that acts to promote aspartate excretion to counteract the potentially deleterious use of O_2_ as an electron acceptor by aspartate oxidase and fumarate reductase, which are major sources of the reactive oxygen species H_2_O_2_ when *E. coli* is transferred from anaerobic to aerobic conditions [22,23]. Thus, it is proposed that YfgF transiently inhibits aspartate taxis or promotes aspartate excretion to lower the production of reactive oxygen species when anaerobic *Salmonella* are rapidly exposed to O_2_ (figure 5). Because the *yfgF* gene is under the control of a Class II FNR-dependent promoter, aerobic inhibition of aspartate taxis would not be readily observed in wild-type bacteria, and thus sustained expression of *yfgF* has allowed this normally transient behaviour to be revealed. Aerobic inhibition of aspartate chemotactic ring formation was observed when the *nuo* operon (encoding the proton translocating NADH dehydrogenase I of the ETC) of *E. coli* was disrupted [13]. However, when supplied with glycerol or combined with a *tsr* mutation the *nuo* mutants did form the aspartate chemotactic ring, suggesting that simultaneous consumption of aspartate and another carbon source was necessary for the formation of the aspartate chemotactic ring [13]. The perturbation of cellular redox state by the *nuo* mutation apparently prevented such conditions being met, resulting in inhibition of aspartate taxis [13]. Thus, the possibility that YfgF acts to inhibit the utilization of an ancillary carbon source that is required for the formation of the aspartate ring on semi-solid agar cannot be excluded, but this possibility is unlikely because YfgF inhibits migration towards a source of aspartate under aerobic conditions in the absence of other carbon sources (figure 2).

MASE1 domains are membrane spanning units that are present in 462 bacterial species, including 164 species of Enterobacteriaceae [26]. These domains are often linked to other domains involved in signalling pathways, including histidine kinases, PAS, GAF, GGDEF and EAL domains [4]. While most MASE1 domains are linked to signalling units, there are examples of ‘stand-alone’ MASE1 domains in 20 bacterial genera [26], suggesting that they can also function independently to sense and transduce signals. The widespread distribution of MASE1 domains suggests that they influence many aspects of bacterial physiology, but this is the first time to the authors' knowledge that a function of a MASE1 domain has been assigned. Future work should be directed towards establishing the precise mechanisms of signal perception and transduction by the YfgF MASE1 domain.

In conclusion, analysis of YfgF shows that it regulates bacterial motility by c-di-GMP-dependent and -independent mechanisms. Cyclic-di-GMP-dependent YfgF activity requires a catalytically active EAL domain, and thus YfgF is an important component in the complex array of interacting DGCs and PDEs that control several aspects of bacterial behaviour. Cyclic-di-GMP-independent YfgF activity is mediated by its MASE1 domain, revealing for the first time the function of a MASE1 domain as a redox-responsive regulator of aspartate taxis.
